# Unfulfilled rehabilitation needs and dissatisfaction with care 12 months after a stroke: an explorative observational study

**DOI:** 10.1186/1471-2377-12-40

**Published:** 2012-06-18

**Authors:** Malin Tistad, Kerstin Tham, Lena von Koch, Charlotte Ytterberg

**Affiliations:** 1 Division of Occupational Therapy, Department of Neurobiology Care Sciences and Society, Karolinska Institutet, Fack 23 200, S 141 83, Huddinge, Sweden; 2 Department of Clinical Neuroscience, Division of Neurology, Karolinska Institutet; Karolinska University Hospital, Stockholm, Sweden; 3 School of Health and Social Studies, Dalarna University, Falun, Sweden; 4 Department of Occupational Therapy, Karolinska University Hospital, Stockholm, Sweden; 5 Department of Neurobiology, Care Sciences and Society, Division of Physiotherapy, Karolinska Institutet, Stockholm, Sweden

**Keywords:** Stroke, Needs, Patient satisfaction, Rehabilitation, Health care services, Long-term

## Abstract

**Background:**

People who have suffered a stroke commonly report unfulfilled need for rehabilitation. Using a model of patient satisfaction, we examined characteristics in individuals that at 3 months after stroke predicted, or at 12 months were associated with unmet need for rehabilitation or dissatisfaction with health care services at 12 months after stroke.

**Methods:**

The participants (n = 175) received care at the stroke units at the Karolinska University Hospital, Sweden. The dependent variables “unfulfilled needs for rehabilitation” and “dissatisfaction with care” were collected using a questionnaire. Stroke severity, domains of the Stroke Impact Scale (SIS), the Sense of Coherence scale (SOC) and socio demographic factors were used as independent variables in four logistic regression analyses.

**Results:**

Unfulfilled needs for rehabilitation at 12 months were predicted by strength (SIS) (odds ratio (OR) 7.05) at three months, and associated with hand function (SIS) (OR 4.38) and poor self-rated recovery (SIS) (OR 2.46) at 12 months. Dissatisfaction with care was predicted by SOC (OR 4.18) and participation (SIS) (OR 3.78), and associated with SOC (OR 3.63) and strength (SIS) (OR 3.08).

**Conclusions:**

Thirty-three percent of the participants reported unmet needs for rehabilitation and fourteen percent were dissatisfied with the care received. In order to attend to rehabilitation needs when they arise, rehabilitation services may need to be more flexible in terms of when rehabilitation is provided. Long term services with scheduled re-assessments and with more emphasis on understanding the experiences of both the patients and their social networks might better be able to provide services that attend to patients’ needs and aid peoples’ reorientation; this would apply particularly to those with poor coping capacity.

## Background

People who have suffered a stroke report needs for health care services that are to a large extent unmet. This applies particularly to rehabilitation and there have been reports of unmet needs for rehabilitation up to eight years after the stroke incident [1-7]. However there is a lack of knowledge about what issues underlie the many unmet needs. The Swedish authorities have adopted a policy of a patient-centred approach in the health care services [8] and this is also included in the National Guidelines for Stroke Care. Patient-centredness is, by the National Board of Health and Welfare [8], described as sensitivity to the patients’ needs, expectations and values. According to the regulations, quality management is also obliged to create prerequisites for patient satisfaction [9,10]. Since patients who are satisfied with the health care services are more likely to be positive about their situation, it is important to achieve patient satisfaction. Such patients, take a more active part in their recommended treatment [11] and it has been suggested that they have a better health outcome [11,12]. More knowledge about needs for rehabilitation from the perspective of the patients and factors related to patient satisfaction is thus imperative.

People with severe disability after a stroke tend to report more unmet needs for rehabilitation [6,7] and are less satisfied [13,14] than people with mild to moderate disability. Disability has, however, been assessed in rather global ways using composite measurements and there is a lack of knowledge about specific aspects of disability that are related to unmet needs for rehabilitation or dissatisfaction with health care services. Further, we have not found any studies in which patient-centred outcome measures have been used as a tool for needs assessment among people with stroke.

Strassers’s comprehensive model of patient satisfaction includes the health care experience as well as characteristics in individuals structured according to socio demographic factors, health status and personality [15]. According to this model, the way patient satisfaction is formed is individual and influenced by socio demographic factors, personality and health status. Using this model as a theoretical framework, the purpose of this study was to generate knowledge about characteristics in individuals that at 3 and 12 months after stroke contribute to unfulfilled needs for rehabilitation or dissatisfaction with health care services at 12 months after stroke.

## Method

### Patient selection and procedures

The data for this study were collected in the context of a prospective observational study of the rehabilitation process after stroke; Life After Stroke phase 1 (LAS 1). All the patients with stroke admitted to the stroke units at Karolinska University Hospital in Huddinge and Solna between May 15, 2006 and May 14, 2007 were eligible for the LAS 1 and 349 patients were included consecutively.

The participants in this study were patients included in LAS-1 who also fulfilled the following criteria; living in the community 3 and 12 months post stroke and having answered at least one of the two questions concerning fulfillment of needs for rehabilitation and satisfaction with care at 12 months. A further criterion was that the follow-ups were completed by the participants themselves although assistance from someone else was allowed.

Upon discharge from the stroke unit, the participants received the services available in that area at the time of the study. This could mean rehabilitation at in-patient facilities and/or specialized day-care rehabilitation, rehabilitation at primary care facilities or home-based rehabilitation or no further rehabilitation.

The study was approved by the Regional Ethical Review Board in Stockholm, Sweden.

### Data collection

Data were collected in the form of a structured face-to-face interview performed by an occupational therapist or a physiotherapist trained for the purpose. Following upon informed consent by the patient, the baseline assessment was carried out at the stroke unit. Remaining data-collection, at three and 12 months post stroke, was carried out in the participants’ home. Information about the participants’ current health condition and impairments were extracted from their medical records.

Using a questionnaire previously used in research exploring neurological disabilities [16-18], data regarding participants’ need for and satisfaction with the health care services received was collected at 12 months after stroke. The questionnaire is based on a taxonomy developed by Ware [19] and covers different dimensions that are thought to influence patients’ satisfaction with care. Levels of agreement concerning 14 statements relating to the different dimensions were rated by the patients on a five-graded Likert scale with “agree” and “do not agree at all” as the endpoints. Two statements were chosen as dependent variables for this study. The dependent variable “unfulfilled needs for rehabilitation” was represented by the statement: “I have received too little rehabilitation after my stroke”. The scores on the statements were dichotomized into fulfilled needs (4–5 on the Likert scale) or unfulfilled needs (1–3). The dependent variable “not satisfied with care” was represented by the statement “I am very satisfied with the care I have received”. The scores were dichotomized into satisfied (1–2) or not satisfied (3–5). Not satisfied will henceforth be referred to as “dissatisfied”.

Taking the point of departure in Strasser’s comprehensive model of patient satisfaction, socio demographic factors were defined as age, sex, civil status, personal finances and education whereas the Barthel Index (BI) and the Stroke Impact Scale 3.0 (SIS) represented aspects of the health status. In the Strasser model, personality is described as a person’s attributes and background. Way of coping can be considered as one aspect of a person’s particular background [20] and represented personality in this study. Way of coping was operationalized as Sense of Coherence (SOC). Table 1 shows the categorization of the independent variables.

**Table 1:** Categorization of the independent variables

**Independent variables**	**Criteria for categorization**
Age	≤65/>65
Sex	Male/female
Civil status	Living with a partner/living alone
Personal finances	Affluent/not affluent
Education	≤9 years/>9 years
Severity	Mild/Moderate or severe
Sense of Coherence (SOC)	Strong SOC (>lowest quartile)/weak SOC (≤lowest quartile)
Stroke Impact Scale (SIS)domains^*^	
Strength	All domains of the SIS:
Memory	Low impact (≥median)/high impact (<median)
Emotion	
Communication	
ADL	
Mobility	
Hand function	
Participation	
Recovery	Good recovery (≥ median)/poor recovery (< median)

^*^SIS at 3 months was used for prediction of unfulfilled needs for rehabilitation/not being satisfied with care. SIS at 12 months was used for association with unfulfilled needs for rehabilitation/not being satisfied with care.

The BI [21], collected at baseline, was used to categorize stroke severity as mild, moderate or severe [22].

The SIS [23], collected at 3 and 12 months post stroke, is considered to be a patient-centred outcome measure [24] and assesses the perceived impact of stroke within eight domains; strength (on the affected side), hand function (on the affected side), mobility, activities of daily living(ADL)/instrumental ADL (IADL), memory, communication, emotion and participation. Each domain contains four to ten items that are rated on a five-graded scale. The score from each domain is transformed to a score between 0–100 for each domain where a score of 100 indicates that there is no impact from the stroke. Further, the SIS contains a recovery-scale on which the patient is asked to indicate how much he or she has recovered between 1 (not recovered at all) and 100 (completely recovered).

The SOC-scale [25,26], collected at 12 months, is considered to measure how people cope with stress-factors they are confronted with. The 13-item version contains statements concerning the three dimensions: comprehensibility; manageability and meaningfulness that are rated on a seven-graded Likert scale. The score range is between 13 (weak SOC) and 91 (strong SOC).

### Analyses

Descriptive statistics were used to present socio-demographic data, medical information, results from the BI, the SIS and the SOC, the frequency of participants with fulfilled/unfulfilled needs for rehabilitation and the frequency of participants satisfied/dissatisfied with care. Proceeded by univariate analyses, four different logistic regression analyses were performed in order to explore the predictive capacity of characteristics in individuals at three months and their association at 12 months with unfulfilled needs for rehabilitation and dissatisfaction with care respectively.

The choice of independent variables for the logistic regressions was based on Strasser’s theoretical model and on the univariate analyses. The standard Enter method by SPSS was used to select the most appropriate model. Significance level was specified at 0.05 and all the statistical analyses were performed using SPSS 17.0 statistical software.

## Result

A total of 175 participants from the LAS-1 fulfilled the inclusion criteria for this study. Reasons for people not being eligible for inclusion were; they lived in a nursing home (n = 33), they answered by proxy (n = 17), an answer on the satisfaction questionnaire was missing (n = 1), they were still at in-rehabilitation at 3 months (n = 2) or deceased (n = 55). Sixty-six participants were lost to follow up because they could not be reached (n = 9), declined to participate (n = 42) or were lost to follow-up for other reasons (n = 15). A comparison between those included in the study and those lost to follow up showed that those lost to follow up did not differ regarding sex (p = 0.12) or age (p = 0.14) but they had had a more severe stroke (p = 0.03).

Table 2 shows medical information, socio demographic factors and scores on the SIS, the BI, the SOC and results from the answers to the questions about needs for rehabilitation and satisfaction with care.

**Table 2:** Baseline characteristics and score on barthel index, stroke impact scale, sense of coherence and the satisfaction questionnaire

	**Baseline n=175 (%)**	**3 months n=170**	**12 months n=175**
Sex male/female	102 (58)/73 (42)		
Age mean (SD) range	68 (14) 24-93		
Civil status, living together/alone	109 (63)/64 (37)*		
Personal finances, Affluent/not affluent	89 (58)/65 (42)^†^		
Education, >9 year/≤ 9 year	96 (56)/74 (44)^‡^		
Diagnosis infarction/haemorrhage	148 (85)/27 (15)		
Localization right/left/both/brainstem/cerebellum/unclear	74 (42)/77 (44)/2 (1)/5 (3)/13 (7)/4 (2))		
Stroke before	47 (27)		
TIA	11 (6)		
Hypertension	100 (57)		
Diabetes	38 (22)		
Severity mild/moderate/severe	144 (82)/26 (15)/5 (3)		
Barthel Index, median (quartiles)	90 (60,100) ^‡^	100 (95,100)^§^	100 (95,100)
Stroke Impact Scale, median (quartiles)			
Strength		75 (62.5,100)^∥^	75 (62.5,100)^#^
Memory		89 (79,96)^§^	91 (79,97)^#^
Emotion		78 (67,86)^**^	83 (67,94)*
Communication		94 (82,100)^††^	96 (86,100)^#^
ADL/IADL		87.5 (71,97.5)^††^	90 (75,100)^#^
Mobility		91 (78,100)^‡‡^	92 (72,100)^#^
Hand function		85 (60,100)^§§^	87.5 (62.5,100)^∥∥^
Participation		75 (54,91)^∥^	81 (59,100)^#^
Recovery		70 (50,85)^‡‡^	75 (57,90)^∥∥^
Sense of Coherence median (quartiles)			78 (68,85)^##^
Satisfaction questionnaire			
Fulfilled needs for rehabilitation			
fulfilled/not fulfilled			116 (67)/58(33)^#^
Satisfaction with care satisfied/dissatisfied			149 (86)/25(14)^#^

*n = 173 ^†^n = 154 ^‡^n = 170 ^§^n = 169 ^∥^n = 166 ^#^n = 174 ^**^n = 164 ^††^n = 168 ^‡‡^n = 167 ^§§^n = 163 ^∥∥^n = 172 ^##^n=158.

### Unfulfilled needs for rehabilitation

Unfulfilled needs for rehabilitation at 12 months after stroke were predicted by high impact in the SIS domain strength (OR 7.05) at three months (Table 3). High impact on the SIS domains hand function (OR 4.38) and poor self-rated recovery (OR 2.46) were associated with unfulfilled needs for rehabilitation at 12 months after stroke (Table 4).

**Table 3:** Multivariate logistic regression regarding factors that at 3 months after stroke predicted unfulfilled needs for rehabilitation at 12 months

**Variables**	**Dichotomisation**	**N**	**OR**	**P-value**	**Confidence intervals**
Strength	Low impact	93			
	High impact	72	7.05	<0.001	3.38-14.7

**Table 4:** Multivariate logistic regression regarding factors that at 12 months after stroke were associated with unfulfilled needs for rehabilitation at 12 months

**Variables**	**Dichotomisation**	**N**	**OR**	**P-value**	**Confidence intervals**
Hand function	Low impact	86			
	High impact	85	4.38	<0.001	2.03-9.48
Recovery	Good recovery	90			
	Poor recovery	81	2.46	0.018	1.16-5.19

### Dissatisfaction with care

Dissatisfaction with care at 12 months after stroke was predicted by a weak SOC (OR 4.18) and high impact on the SIS domain participation (OR 3.78) at three months (Table 5). A weak SOC (OR 3.63) and high impact on the SIS domain strength (OR 3.08) were associated with not being satisfied with care at 12 months (Table 6).

**Table 5:** Multivariate logistic regression regarding factors that at 3 months after stroke predicted dissatisfaction with care at 12 months

**Variables**	**Dichotomisation**	**N**	**OR**	**P-value**	**Confidence intervals**
Participation	Low impact	90			
	High impact	75	3.78	0.018	1.25-11.39
Sense of Coherence	Strong	120			
	Weak	37	4.18	0.006	1.52-11.53

**Table 6:** Multivariate logistic regression regarding factors that at 12 months after stroke were associated with dissatisfaction with care at 12 months

**Variables**	**Dichotomisation**	**N**	**OR**	**P-value**	**Confidence intervals**
Strength	Low impact	111			
	High impact	62	3.714	0.023	1.66-8.14
Sense of Coherence	Strong	120			
	Weak	37	6.63	0.009	1.38-9.59

## Discussion

This study examined characteristics in individuals that contribute to self-reported unfulfilled needs for rehabilitation or dissatisfaction with care after stroke. Using Strassers’s model of patient satisfaction, unfulfilled needs for rehabilitation were only predicted by, or associated with factors considered as aspects of the patients’ health status. The findings suggest a presence of unmet needs related to disabilities that are common after stroke and consequently that there probably are incentives for further rehabilitation beyond the initial phase after stroke. Dissatisfaction with care was predicted by and/or associated with factors derived from both health status and personality that are included in Strasser’s model. The results confirm that patient satisfaction is a multidimensional construct [11,15] and indicate that health care services after stroke are less tailored to the demands of people with poor ability to cope with stressful situations.

Using a patient-centred outcome measure, we found that high impact on the SIS domain strength predicted unfulfilled need for rehabilitation and high impact on hand function was associated with unfulfilled needs for rehabilitation. This is in line with previous studies that have shown that people with severe disability according to the Rankin Scale are more likely to report unmet needs for therapy [6]. In contrast to the results from the present study, Kersten et al. reported more unmet needs for rehabilitation among people with poor mobility. A reason for the disparate results might be the different perspectives on mobility in the two studies: Kersten et al. related their findings to the ability to walk 10 meters [7] whereas the SIS domain mobility used in this study addresses the self-perceived impact of stroke on different aspects of mobility. Disabilities related to strength and hand function as well as to mobility are, however, common after stroke; and even if interventions have been directed toward these disabilities, the patients may have wanted further rehabilitation. Physiotherapy has been regarded as a means to physical recovery [27-30] and people may consider that they have potential for further recovery if more is provided. Moreover, strategies taught at the rehabilitation facilities are far from always transferrable to the patients’ daily life [31,32] and consequently there might be aspects of these problems that do not surface until the patients return home.

Poor self-rated recovery was associated with unfulfilled needs for rehabilitation at 12 months after stroke. A qualitative study indicated that the patients’ understanding of recovery is “full recovery” and to taking up the same activities as before their stroke [29]. There have been descriptions of high expectations for physiotherapy as a method to achieve recovery and moreover as something that represents faith and hope for many patients [30]. Furthermore, patients might not have been confronted with their limited potential for further recovery at the time of their discharge from rehabilitation, which may delay the process of adaptation [28]. The absence of full recovery might consequently contribute to reports of unfulfilled needs for rehabilitation. A part of the goal for long term rehabilitation after stroke is “to help the person to make the best adaptation possible to any difference between roles achieved and roles desired” [33]. One way of contributing to that goal might be to attend to the patients’ self-rated recovery in relation to the recovery they expect and moreover to develop interventions in rehabilitation that have the potential to help people modify unrealistic expectations.

A weak SOC was of importance for being dissatisfied with care in this study. This result is consistent with findings reported by Larsson who suggested that a weak SOC was related to dissatisfaction with care [34]. A weak SOC is thought to be related to poor ability to mobilize emotional, intra- and interpersonal resources as well as material resources to cope with a problem. It also includes the abilities of the person’s social network as potential resources [25]. The findings in this study then suggest that the health care services are preferentially tailored to the demands of people with a good ability to cope with stressful situations, and people with a weak SOC might thus be in need of extra or different support from the health care services. A patient-centred approach in health care services for stroke survivors emphasizes the need to understand the experiences of both the patients, and their carers, and should also support the family and/or the social network of people who have had a stroke [24]. In order to reach those in greatest need, patient-centred services that offer scheduled long-term re-assessments might well be of additional value to people with a weak SOC.

The strengths of the study are its patient-centred perspective, represented by the SIS, and the fact that proxy-answers were not included. However, even though the SIS covers a substantial part of the problems perceived as important after a stroke, it does not cover e.g. fatigue which is one of the most commonly reported problems after a stroke [35]. Consequently, there may be important problems that are not taken into consideration in this study but which contribute to unfulfilled needs for rehabilitation and patients not being satisfied with care. Since the participants lost to follow-up had more severe stroke, some caution should be exercised in the interpretation of the findings as the sample may not be quite representative for people with the most severe stroke who were living at home one year after a stroke. Further, 17 participants did not have a SOC score and were not included in logistic regression analyses in models with SOC.

This study takes factors that characterize the patient: socio demographic factors, health status and personality, into consideration. It has, however, been shown that the provision of health care services beyond the initial phase affects satisfaction with care and fulfillment of rehabilitation needs among patients with stroke independent of patient characteristics [14]. Further studies should pay also attention to the provision of health care services; this should be the case both with regard to the amount of rehabilitation received and also at what point in time in the process following a stroke the services are provided.

## Conclusions

Among people who had suffered a stroke and who lived in the community, 33% reported unmet needs of rehabilitation and 14% were dissatisfied with the health care received the first year after stroke. Unfulfilled needs for rehabilitation were associated with different aspects of the impact from stroke, whereas dissatisfaction with care was associated with impact from stroke as well as personality. Thus, since people may perceive further potential for recovery even after the relatively short period in which rehabilitation usually is provided, and since new problems may arise when people gradually try to regain their former life-situation or adjust to a new one, there is a need for the development of patient-centred long-term rehabilitation services beyond the initial phase of recovery. In order to attend to the needs for rehabilitation among the people with stroke when the needs arise, more flexibility in terms of when the rehabilitation is provided is consequently required in rehabilitation services. Further long-term health care services should be able to provide care in a way that helps people with various individual characteristics e.g. poor coping ability to adapt to and to handle a new life situation. In order to optimize the allocation of health care resources, the provision of rehabilitation and other health care services needs to be targeted towards those in need of services. The results from this study provide clinically relevant knowledge regarding aspects of the individual characteristics of patients that could be monitored with the aim of targeting long-term rehabilitation and other health care services.

## Competing interests

The authors declare that they have no competing interests.

## Authors’ contribution

MT participated in the design of the study, performed the analysis and drafted the manuscript. KT participated in the design of the study and made critical revision of the manuscript. LvK participated in the design of the study, acquired the data, participated in analysis and interpretation of the data and made critical revision of the manuscript. CY participated in the design of the study, in analysis and interpretation of the data and made critical revision of the manuscript. All authors have read and approved the final manuscript.

## Pre-publication history

The pre-publication history for this paper can be accessed here:

http://www.biomedcentral.com/1471-2377/12/40/prepub
